# Efficacy and Safety of Intra-Class Switching from Ixekizumab to Secukinumab in Patients with Plaque Psoriasis: A Multicenter Retrospective Study

**DOI:** 10.3390/jpm14121169

**Published:** 2024-12-22

**Authors:** Nicoletta Bernardini, Annunziata Dattola, Raimondo Rossi, Gianluca Pagnanelli, Paolo Amerio, Laura Atzori, Cristina Mugheddu, Viviana Lora, Domenico Giordano, Lucia Finistauri Guacci, Severino Persechino, Antonio Giovanni Richetta, Nevena Skroza, Concetta Potenza

**Affiliations:** 1 Dermatology Unit “Daniele Innocenzi”, ASL Latina, Department of Medico-Surgical Sciences and Biotechnologies, Sapienza University of Rome, 04100 Latina, Italy; nevena.skroza@uniroma1.it (N.S.); concetta.potenza@uniroma1.it (C.P.); 2 Dermatology Clinic, Department of Clinical Internal, Anesthesiological and Cardiovascular Sciences, Sapienza University of Rome, 00185 Rome, Italy; nancydattola@gmail.com (A.D.); raimondo.rossi@uniroma1.it (R.R.); finistauriguacci.1697680@studenti.uniroma1.it (L.F.G.); antonio.richetta@uniroma1.it (A.G.R.); 3 1st Dermatology Division, Dermopathic Institute of the Immaculate (IDI-IRCCS), 00167 Rome, Italy; g.pagnanelli@idi.it; 4 Section of Dermatology, Department of Medicine and Aging Science, G. D’Annunzio University, 66100 Chieti, Italy; p.amerio@unich.it; 5 Department Medical Sciences and Public Health, University of Cagliari, 09124 Cagliari, Italy; atzoril@unica.it (L.A.); cristinamugheddu@tiscali.it (C.M.); 6 Division of Dermatology, San Gallicano Dermatological Institute, 00144 Rome, Italy; viviana.lora@ifo.it; 7 Department of Neurosciences, Mental Health and Sensory Organs, Sapienza University, 00185 Rome, Italy; domenico.giordano@uniroma1.it (D.G.); severino.persechino@uniroma1.it (S.P.)

**Keywords:** secukinumab, ixekizumab, switch, psoriasis, psoriatic arthritis, intra-class, hidradenitis suppurativa, PASI-90

## Abstract

**Background:** the present multicenter retrospective study aimed to evaluate the efficacy and safety of intra-class switching between interleukin-17A (IL-17A) inhibitors, specifically from ixekizumab to secukinumab, in patients with plaque psoriasis. **Methods:** this study included 11 patients (6 male, 5 female) who had previously received ixekizumab and then were switched to secukinumab. Patients’ PASI, DLQI, and pain VAS (in those with psoriatic arthritis) were evaluated at weeks 16, 24, 54, and 98. **Results:** PASI-90 was reached in 10 (91%) cases at week 24. One patient experienced secondary failure to secukinumab at week 98. No adverse events were reported. **Conclusions:** overall, the majority of patients experienced a favorable response to secukinumab, suggesting that it may be an effective treatment option for patients with an inadequate response or loss of efficacy to ixekizumab.

## 1. Introduction

Plaque psoriasis is a chronic, immune-mediated inflammatory disorder of the skin characterized by erythematous, scaly, and itchy plaques; it can be associated with joint involvement, known as psoriatic arthritis, which presents in various clinical forms, including asymmetric oligoarthritis, symmetric polyarthritis, and spondylitis. Other variants of psoriasis exist, such as the guttate form, the pustular form, and the erythrodermic form, which are less common and are distinct from plaque psoriasis in terms of genetic background, clinical presentation, and treatment response. This disease often has a significant impact on patients’ quality of life and is associated with an increased risk of comorbidities, such as cardiovascular disease and depression [1].

Psoriasis has a worldwide prevalence of 1–3% in the adult population and is more common in high-income countries with older populations [2]. The incidence ranges from 0.03% to 0.32% and shows two peaks: the first around 20 years of age and the second around 60 years [2]. 

Its pathogenesis is characterized by a complex interplay of genetic, environmental, and immunological factors [1]. Certain genes predispose individuals to the onset of psoriasis, and genome-wide association studies have identified nine chromosomal regions that increase susceptibility, including PSORS1 located on chromosome 6p [3]. Based on genetic susceptibility, physical and psychological stressors trigger the initiation of an inflammatory cascade involving several cytokines, such as tumor necrosis factor α (TNFα), interleukin-12, interleukin-17, and interleukin-23. 

The advent of biologic therapies that target the specific cytokines involved in the inflammatory pathway has transformed the treatment landscape of psoriasis. The first biologic drugs introduced were TNFα inhibitors, particularly infliximab, which was serendipitously observed to be effective against psoriasis in a patient with Crohn’s disease [4]. Subsequently, the role of other cytokines, including interleukin-17, was clarified [5], leading to the development of newer biologic agents. Among these biologic agents approved for the treatment of plaque psoriasis are the interleukin-17A (IL-17A) inhibitors, such as secukinumab and ixekizumab; both have demonstrated significant efficacy in terms of reduction in Psoriasis Area and Severity Index (PASI) and Dermatology Life Quality Index (DLQI) and have generally exhibited a favorable safety profile [6,7,8]. Ixekizumab, the more recently approved agent, entered the market in April 2016 [9], while secukinumab received regulatory approval in January 2015 [10].

While there is established evidence on the efficacy and safety of inter-class and intra-class switching [11,12,13,14] and growing evidence, particularly, on the switch from secukinumab to ixekizumab in plaque psoriasis and psoriatic arthritis [13,15,16,17,18,19,20,21,22], data regarding the transition from ixekizumab to secukinumab are lacking. To address this gap and to prove that, the present multicenter retrospective study was conducted to evaluate the efficacy and safety of switching from ixekizumab to secukinumab in patients with plaque psoriasis.

## 2. Materials and Methods

**Figure 1:**
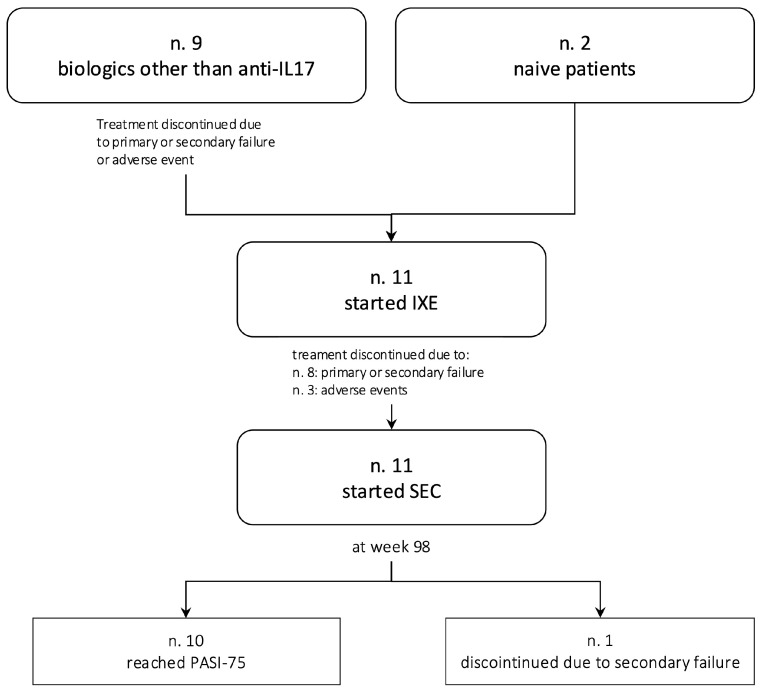
Flowchart summarizing the allocation of patients throughout the various phases of the study. Anti-IL17: anti-interleukin 17; IXE: ixekizumab; SEC: secukinumab.

This retrospective study collected data across several Italian centers and included 11 patients with plaque psoriasis, either with or without psoriatic arthritis, who had previously received ixekizumab 80 mg every 4 weeks and were subsequently switched to secukinumab 300 mg every 4 weeks because of primary or secondary failure or an adverse event to the former (Figure 1). After the switch, during each outpatient visit, every center assessed specific outcomes on disease severity and quality of life utilizing, respectively, PASI and DLQI assessment scales. PASI is a severity scoring system used in plaque psoriasis in which lesions’ erythema, scaling, and thickness are graded for each body region; it is widely employed in clinical trials and routine practice. DLQI is a simple and reliable patient-reported quality-of-life measure developed by Finlay et al. that is routinely utilized in clinical practice and studies [23]. Furthermore, patients were monitored for adverse events after the switch to assess therapy safety.

Detailed data on patient demographics, disease characteristics, and prior treatments were collected from medical records, in particular: age, gender, weight, height, BMI, concurrent psoriatic arthritis, comorbidities, prior therapy with other biologic drugs and ixekizumab, its duration and reason for discontinuation, efficacy outcomes, and safety events.

Patients’ PASI, DLQI, and pain Visual Analog Scale (VAS) in those with psoriatic arthritis were evaluated at baseline, week 16, 24, 54, and 98; treatment success was defined as achieving a 90% or greater reduction PASI from baseline (PASI-90) at 24 weeks; treatment was continued if PASI-50 was reached and the DLQI was ≤5 after 16 weeks [24]. Concurrent topical therapies, such as corticosteroids, vitamin D analogs, and salicylic acid or urea-based emollients, were permitted. 

All patients signed an informed consent form as per the ethics committee requirement.

Inclusion criteria were as follows: patients aged 18 years or older; moderate-to-severe psoriasis (PASI > 10) and/or (DLQI > 10); previous therapy with ixekizumab 80 mg with subsequent primary or secondary failure or an adverse event leading to discontinuation of treatment; patients who experienced primary or secondary failure with other biologic drugs before ixekizumab were also included. Exclusion criteria were patients younger than 18 years, mild disease (PASI ≤ 10 and/DLQI ≤ 10), and those who had not received prior treatment with ixekizumab.

### Patient Demographics and Clinical Profiles

Patient 1: A 69-year-old female with psoriatic arthritis and comorbidities, including arterial hypertension, dyslipidemia, autoimmune thyroiditis, and diabetes mellitus. She had previously been treated with etanercept and adalimumab, both discontinued due to secondary treatment failure, before switching to ixekizumab.

Patient 2: An 80-year-old female with psoriatic arthritis, arterial hypertension, dyslipidemia, and diabetes. She had initially been treated with etanercept, which was discontinued due to an adverse event, followed by ustekinumab, which was discontinued due to secondary failure, before initiating ixekizumab.

Patient 3: A 71-year-old female with arterial hypertension who was biologic-naïve before starting ixekizumab.

Patient 4: A 36-year-old male with arterial hypertension and dyslipidemia, who was previously treated with adalimumab, which was discontinued due to secondary failure.

Patient 5: A 60-year-old male with psoriatic arthritis and arterial hypertension, previously treated with adalimumab, which was discontinued due to primary failure.

Patient 6: A 56-year-old male with psoriatic arthritis and dyslipidemia who had been treated with etanercept, adalimumab, and ustekinumab, all of which were discontinued due to treatment failure.

Patient 7: An 18-year-old female who was biologic-naïve before starting ixekizumab.

Patient 8: A 62-year-old male with arterial hypertension, previously treated with infliximab (discontinued due to secondary failure) and ustekinumab. He was lost to follow-up but returned in June 2019 with worsening symptoms and was then started on ixekizumab.

Patient 9: A 65-year-old male with hypercholesterolemia, previously treated with etanercept, which was discontinued due to secondary failure.

Patient 10: A 34-year-old female with psoriatic arthritis, obesity, dyslipidemia, and fatty liver disease, previously treated with adalimumab.

Patient 11: A 36-year-old male with psoriatic arthritis as his primary comorbidity, previously treated with adalimumab, discontinued due to secondary failure.

## 3. Results

The demographics and clinical outcomes of the 11 patients who met the inclusion criteria are summarized in Table 1.

This study included a total of 11 patients (6 male, 5 female); the mean age was 53.35 years (SD = 19.38), while the mean BMI was 27.84 (SD = 5.75); 6 patients had concomitant psoriatic arthritis; the most common comorbidities were arterial hypertension and dyslipidemia and/or hypercholesterolemia.

Prior to the treatment with ixekizumab, nine patients were treated with other biologics (adalimumab, etanercept, infliximab, and ustekinumab); the reason for discontinuation in this sub-group was mostly secondary failure, while only patient 2 developed an adverse event to etanercept. Two patients (patient 3 and patient 7) were treatment-naive and started ixekizumab 80 mg as the first biologic treatment. The reason for the discontinuation of ixekizumab was a failure (either primary or secondary) in eight patients. In the remaining three cases, adverse events were reported: patient 8 developed hidradenitis suppurativa, patient 10 experienced an injection site reaction after 40 weeks of therapy, and patient 11 was affected by recurrent fungal infections attributed to ixekizumab. In total, 10 out of 11 patients had received ixekizumab therapy for more than 12 months prior to switching to secukinumab 300 mg; patient 3 received ixekizumab for 6 months and was switched due to lack of efficacy (primary failure).

**Figure 2:**
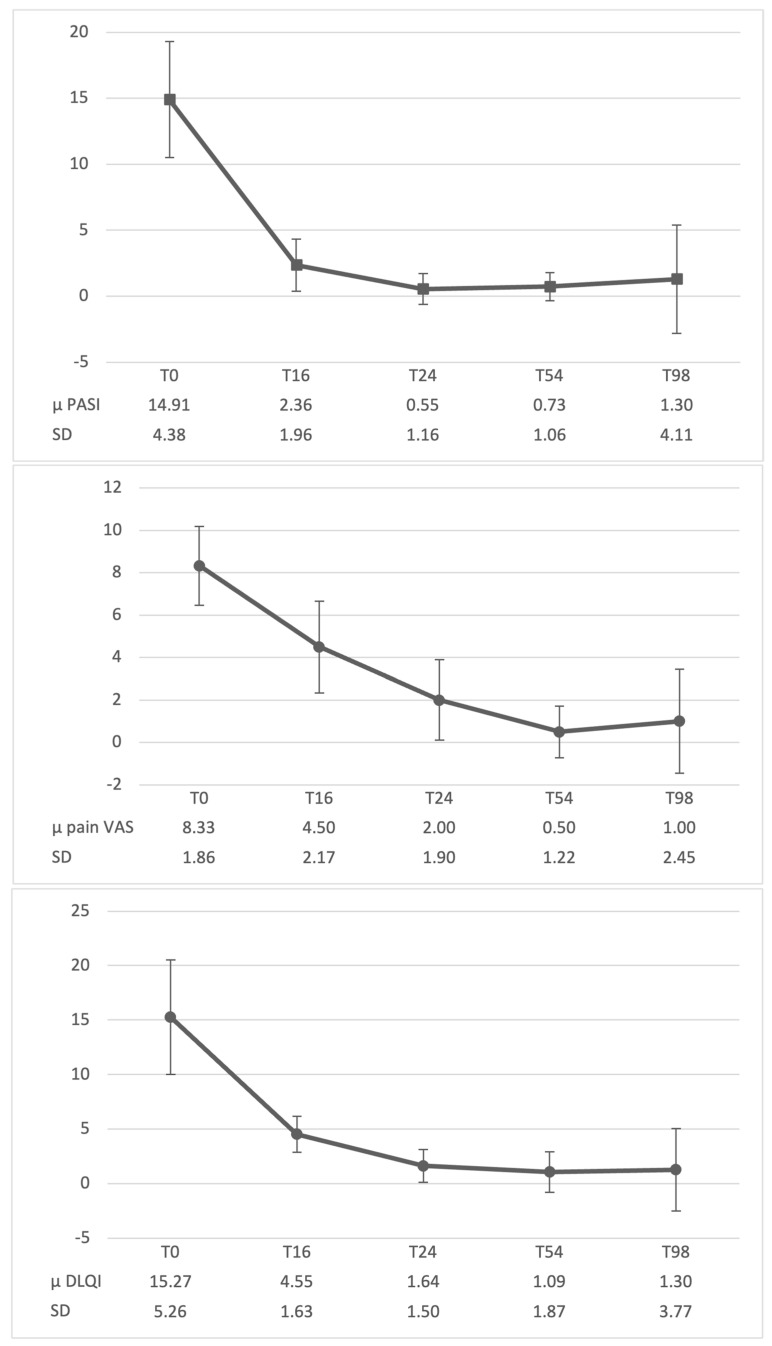
Mean PASI, DLQI and pain VAS trend throughout the study period. μ: mean; PASI: psoriasis Area and Severity Index; DLQI: Dermatology Life Quality Index; VAS: Visual Analog Scale; SD: standard deviation.

All patients treated with secukinumab 300 mg were followed for 98 weeks after the induction dose (Figure 2). At week 16, eight patients (73%) achieved a PASI-75 response, while three patients (27%) reached PASI-50; by week 24, nine patients (82%) reached a PASI-90 response, with two additional patients reaching PASI-75. By the end of the 98-week evaluation, 10 out of 11 patients (91%) had attained a PASI-90 response, while 1 patient experienced secondary treatment failure and was subsequently discontinued from the study. DLQI ≤ 5 was reached by week 16 by nine patients (82%) and at week 24 by all the patients. Furthermore, in patients with concurrent psoriatic arthritis, a marked reduction in pain VAS was observed by week 24, with all patients reporting a VAS score below 5.

## 4. Discussion

The aim of this study was to demonstrate that intra-class switching from the IL-17A inhibitor ixekizumab to the IL-17A inhibitor secukinumab represents a valuable, effective, and safe treatment option for patients who had an inadequate response or loss of efficacy with the former anti-IL-17 agent. To our knowledge, this is the first multicenter retrospective study to address this specific scenario.

Existing studies [13,15,16,17,18,19,20,21,22] have demonstrated that switching between interleukin-17A inhibitors represents a valuable therapeutic option for patients who experience primary or secondary treatment failure or adverse events. While the underlying pathophysiological mechanisms driving the efficacy of this intraclass switch remain unclear, the available clinical data suggests it can lead to a favorable therapeutic response. However, the current literature has primarily focused on the transition from secukinumab to ixekizumab, with limited evidence addressing the reverse switch from ixekizumab to secukinumab.

Secukinumab demonstrated a rapid onset of action, with the majority of patients achieving a PASI 75 response by week 16 and an even greater proportion (82%) reaching PASI 90 by week 24. This substantial clinical improvement was sustained throughout the 98-week evaluation period, and no adverse events were reported. Patient 3 had primary non-response to ixekizumab and managed to achieve a PASI-90 response with secukinumab at week 24, which was maintained through the 98-week evaluation period; this shows that intra-class switching is a viable option even in patients with primary lack of response to the preceding anti-IL17 agent. Only patient 6 suffered a loss of efficacy to secukinumab (secondary failure) at week 98, leading to a change with another biologic drug. Notably, this patient had previously undergone treatment changes since 2013 involving a total of four biologic drugs, including ixekizumab. This places patient 6 within the well-recognized category of patients with difficult-to-treat psoriasis and psoriatic arthritis, characterized by multiple therapeutic switches (multi-failure) throughout their clinical history.

Both patients (patient 3 and patient 7) who were treatment-naïve prior to ixekizumab demonstrated an optimal response, achieving PASI 90 at week 24 and sustaining this response throughout the entire monitoring period.

For patient 8, who experienced hidradenitis suppurativa (HS) while on treatment with ixekizumab, switching to secukinumab was a reasonable choice, as secukinumab, unlike ixekizumab has the approved therapeutic indication for this condition [25] considering the role of IL-17A in the pathogenesis of HS [5]. The patient exhibited a rapid improvement in axillary and inguinal lesions in the following weeks and achieved remission by week 24; by week 98, there were no further disease flare-ups. 

While anti-TNFα agents are also indicated for the treatment of hidradenitis suppurativa, many patients with concomitant plaque psoriasis, as in our study cohort, may have already received prior therapy with these biologics. In such cases, switching to secukinumab, which has a distinct mechanism of action, could be a prudent choice to avoid potential plaque psoriasis flare-ups that may occur with a step-down in treatment to anti-TNFα agents.

This study, nevertheless, has some limitations: its retrospective design, the small sample size, and the inherent subjectivity in the PASI assessment, which could result in variability between different centers. Moreover, due to the small cohort size, the study was not powered for statistical analysis. More studies, with a larger sample size and possibly a longer duration of follow-up, are warranted to further elucidate the long-term efficacy and safety of the intraclass switch from ixekizumab to secukinumab.

This retrospective study suggests that secukinumab could be a valuable treatment option in terms of clinical efficacy, safety, and cost-effectiveness for patients who have experienced an inadequate response or loss of efficacy with the interleukin-17A inhibitor ixekizumab, despite the two agents belonging to the same class. Indeed, we propose that intra-class switching be regarded as an initial strategy prior to transitioning to a biologic of a different class, given the favorable outcomes in efficacy and safety observed. Secukinumab could also be considered a systemic treatment option in case of concurrent plaque psoriasis and hidradenitis suppurativa in patients since its recent approval for managing both conditions.

## Figures and Tables

**Table 1:** Patient characteristics and clinical outcomes after switching from ixekizumab to secukinumab.

Variables	Value
Sex, *n* (%)	
Male	6/11 (54.55)
Female	5/11 (45.45)
Mean age, years ± SD	53.36 ± 19.38
Mean BMI ± SD	27.84 ± 5.75
Comorbidities, *n* (%)	
Arterial hypertension	6/11 (54.55)
Dyslipidaemia and/or hypercholesterolemia	6/11 (54.55)
Diabetes	2/11 (18.18)
Fatty liver disease	1/11 (9.09)
Autoimmune thyroiditis	1/11 (9.09)
Obesity	1/11 (9.09)
Previous treatments with drugs other than IXE, *n* (%)	9/11 (81.81)
Reason for discontinuation of IXE treatment, *n* (%)	
Failure	8/11 (72.72)
Adverse events	3/11 (27.27)
Efficacy of SEC	
Mean interval between start of IXE and switch (months)	13.91 ± 4.74
Mean PASI at baseline	14.91 ± 4.38
Mean Pain VAS at baseline	4.55 ± 4.55
Mean DLQI at baseline	15.27 ± 5.26
≥PASI-90 at week 24, *n* (%)	9/11 (81.81)
DLQI ≤ 5 at week 16 *n* (%)	9/11 (81.81)
Pain VAS ≤ 5 at week 16 *n* (%)	4/6 (66.67)
Reported adverse events, *n* (%)	0/11 (0)

BMI, body mass index; IXE, Ixekizumab; SEC, Secukinumab; PASI-90, 90% reduction in psoriasis area and severity index; DLQI, Dermatology Life Quality Index; Pain VAS, Pain Visual Analog Scale.

## Data Availability

The data presented in this study are available upon reasonable request from the corresponding author.
